# Inhibition of purine nucleoside and nucleobase transporters by tyrosine kinase inhibitors

**DOI:** 10.1371/journal.pone.0348426

**Published:** 2026-05-15

**Authors:** Nayiar Shahid, Chan H. Kim, Kerrylei B. Jabilona, Khanh H. Nguyen, Nicholas M. Ruel, James R. Hammond

**Affiliations:** Department of Pharmacology, Faculty of Medicine & Dentistry, University of Alberta, Edmonton, Alberta, Canada; University of Coimbra: Universidade de Coimbra, PORTUGAL

## Abstract

Tyrosine kinase inhibitors (TKI) are often used in combination with other chemotherapeutic nucleoside/nucleobase analogues, such as gemcitabine and 6-mercaptopurine, in the treatment of various cancers. Past studies have shown that several TKI inhibit the cellular uptake of nucleoside analogues by the equilibrative nucleoside transporter subtype 1 (ENT1), and suggest that TKI may also inhibit other related nucleoside and nucleobase transporters such as ENT2 and the equilibrative nucleobase transporter (ENBT1). To assess this possibility in a controlled manner, we have compared the ability of a series of TKI to inhibit each of these transporters in HEK293 cells that have been genetically modified to express either ENT1, ENT2 or ENBT1 in isolation, and on ENBT1 natively expressed in the chronic myeloid leukemia cell line K562. All TKI tested inhibited ENT1 and ENT2 with K_i_ values ranging from 1 to 30 µM, typically with higher affinities for ENT1 than for ENT2. Gefitinib, which was one of the most effective inhibitors of ENT1, also inhibited ENBT1 with a similar affinity. The loss or gain of these transporters had no impact on the ability of gefitinib to directly affect cell viability, indicating that they were unlikely to be involved in the cellular uptake of the TKI. These data suggest that TKI inhibit multiple purine transporters, likely via interactions with their common purine ring binding domain. However, interactions between TKI and other nucleoside/nucleobase analogue drugs that are substrates for these systems are not likely to be a significant concern at the doses commonly used therapeutically.

## Introduction

Tyrosine kinase inhibitors (TKI) have emerged as an important class of drugs in the field of cancer therapy, offering targeted treatment options for a variety of malignancies, including chronic myeloid leukemia (CML) [1] and Philadelphia chromosome-positive acute lymphoblastic leukemia (Ph^+^ ALL) [2]. These inhibitors work by binding to the tyrosine kinase ATP-binding domain and blocking the phosphorylation of their substrates, thereby preventing the activation of downstream pathways that are crucial for cell growth and proliferation. By disrupting these signaling pathways, TKI effectively impede the proliferation and spread of cancer cells. [3]

While TKI have revolutionized cancer therapy, they are seldom used alone, and are often paired with nucleobase or nucleoside analogue drugs such as 6-mercaptopurine (6-MP) or gemcitabine, respectively. 6-MP and gemcitabine require the activity of nucleobase and nucleoside transporters to enter cells and produce their chemotherapeutic effects [4–6]. It has been reported that TKI can inhibit the cellular uptake of nucleobases via a sodium-independent process in human renal proximal tubule epithelial cells. [7] The molecular identity of the transporter involved was not known at the time of this earlier study, but we hypothesized that it was likely the recently identified equilibrative nucleobase transporter 1 (ENBT1), encoded by *SLC43A3* [8], which has been shown to mediate the transport 6-MP into cells [4,9].

TKI have also been reported to inhibit equilibrative nucleoside transporters, specifically subtype 1 (ENT1) [10–13]; their interaction with the other major subtype of equilibrative nucleoside transporter, ENT2, has not been as clearly established. Given the similarity in substrate preferences of ENT1 and ENT2 [14,15], we hypothesized that TKI should have similar inhibitor affinities for both ENT subtypes, particularly if they are interacting with the substrate binding domain.

Interactions between drugs for these transporter proteins are of potential concern therapeutically, as co-treatment may lead to the inhibition of cellular drug accumulation, such as 6-MP (used in combination with TKI for Ph + ALL) via nucleobase transporters, or the inhibition of chemotherapeutic nucleoside analogues known to be substrates for ENTs, such as gemcitabine. While TKI have been reported to inhibit nucleobase and nucleoside transport, these studies were done using a variety of cell models and methodologies. Furthermore, if TKI not only inhibit purine transporters but are actually substrates for these systems then changes in transporter expression levels may impact the ability of TKI to enter cells and produce their cytotoxic effects via block of receptor tyrosine kinases. The present study was conducted to clarify the relative affinities of TKI for these transporters, and to examine the impact of transporter expression/loss on the cytotoxicity of TKI, using a recently developed panel of genetically modified cells designed to express each of ENBT1, ENT1, and ENT2 in isolation [14,15].

## Methods and materials

### Materials

[8-^3^H]-2-chloroadenosine (1 mCi/ml), [2,8-^3^H]adenine (1 mCi/ml), [^14^C]6-MP (1 mCi/ml) and [^3^H]-water (1 mCi/g) were obtained from Moravek Biochemicals (Brea, CA). Dipyridamole, geneticin, Dulbecco’s Modified Eagle’s Medium (for HEK293 cell culture), Iscove’s Modified Dulbecco’s Medium (for K562 cell culture), fetal bovine serum (FBS), penicillin-streptomycin solution, light mineral oil, adenine, 6-MP, and 2-chloroadenosine were purchased from Sigma-Aldrich (St. Louis, MO). All TKI were purchased from Tocris Bioscience (Toronto, ON). The Ecolite liquid scintillation cocktail was purchased from MP Biomedical (Irvine, CA). Dow DOWSIL™ 550 Silicone Fluid (silicone oil) was from Ellsworth Adhesives Canada.

### Cell culture

HEK293 cells were purchased from ATCC (Manassas, VA). K562 cells were generously provided by Dr David Eisenstat (Department of Oncology, University of Alberta). The ENT1KO, ENT2KO and E1E2KO cell lines (CRISPR modified HEK293 cells), and HEK293 cells stably transfected with *SLC43A3* (HEK293-SLC43A3 cells), were created and characterized as described previously. [4,14,15] All cell lines were cultured at 37°C in a humidified 5% CO_2_ incubator in the presence of penicillin (100 U/ml), streptomycin (100 μg/ml), sodium pyruvate (1 mM), and 10% FBS for HEK293-SLC43A3, K562, ENT1KO and HEK293 cells or 20% FBS for the ENT2KO and E1E2KO cell lines. The HEK293-SLC43A3 cell media additionally contained 120 µg/ml geneticin to maintain selection pressure for the stable transfectant. The kinetics of 2-chloroadenosine transport by HEK293 cells with ENT1 and/or ENT2 deleted (ENT1KO and ENT2KO cells, respectively) were similar to those previously described by us. [14] Likewise, the kinetics of [^3^H]adenine and [^14^C]6-MP transport by HEK293 cells stably transfected with ENBT1 were previously determined in our laboratory. [4,16]

### Nucleoside/nucleobase transport assays

ENT1 and ENT2 activity was assessed by the ability of cells to accumulate [^3^H]2-chloroadenosine. ENBT1 activity was measured based on the cellular accumulation of [^3^H]adenine or [^14^C]6-MP in the presence of the broad-spectrum ENT inhibitor dipyridamole (1 µM). To eliminate the potential contribution of sodium-dependent concentrative nucleoside transporters, cells were suspended in sodium-free buffer (NMG buffer: 140 mM N-methylglucamine, 5 mM KCl, 4.2 mM KHCO_3_, 0.36 mM K_2_HPO_4_, 0.44 mM KH_2_PO_4_, 10 mM HEPES, 0.5 mM MgCl_2_, 1.3 mM CaCl_2_, pH 7.4). Non-mediated uptake of [^3^H]2-chloroadenosine was assessed in the presence of 5 mM adenosine (complete ENT1 and ENT2 block), while the non-mediated uptake of [^14^C]6-MP was assessed in the presence of 5 mM non-radiolabelled adenine. In experiments where [^3^H]adenine was used as the substrate to study ENBT1 activity in the HEK293-SLC43A3 cells, background uptake (non-ENBT1-dependent) was assessed by measuring, in parallel, the cellular association of [^3^H]adenine by un-transfected HEK293 cells. We have previously established that the base (un-transfected) HEK293 cells had negligible levels of ENBT1 activity [4]. To initiate the uptake assay, 250 μl of cell suspension was added to 250 μl of radiolabeled substrate (± test inhibitor) layered over 21:4 (v:v) silicone:mineral oil (200 μl) in microcentrifuge tubes. The uptake reaction was terminated after a defined time by centrifugation of the cells through the oil layer at 10,000 g. The aqueous layer was aspirated and the tube above the oil was washed with approximately 1 ml of NMG buffer. The water and the oil were then sequentially removed, leaving only the cell pellet. The cell pellet was digested in 1 M sodium hydroxide for ~16 h after which the radioactive content of each sample was measured using liquid scintillation spectroscopy. Mediated uptake was defined as the difference between total and non-mediated uptake. K_i_ values were calculated using the Cheng-Prusoff equation [17], based on the IC_50_ values derived from the concentration-response curves, and the K_m_ of the respective substrates for the transporters as reported previously (34 ± 7 μM and 23 ± 5 μM for 2-chloroadenosine transport by ENT1 and ENT2, respectively; 163 ± 63 μM for 6-MP transport by recombinant ENBT1 in HEK293 cells) [4,14,15], or that determined in the present study for 6-MP transport by endogenous ENBT1 in K562 cells.

### Cell viability analysis

Cells were seeded into 24-well plates at a density of 1 x 10^5^ cells per well in complete culture medium. At 1 h following seeding the culture media was supplemented with a range of concentrations of the test compound and cells were then incubated for 48 h at 37°C in a humidified 5% CO_2_ atmosphere (compound-mediated response). To determine the baseline cell viability (100% cell viability), cells were treated with medium containing DMSO only (at the concentration used to dissolve the test compounds). Medium containing the test compound in the absence of cells was used to determine the 0% cell viability (background). After 48 h treatment, the media was removed and replaced with 250 µL of Dulbecco’s phosphate buffered saline (DPBS – 137 mM NaCl, 2.7 mM KCl, 6.3 mM Na_2_HPO_4_, 1.5 mM KH_2_PO_4_, 0.5 mM MgCl_2_, 0.9 mM CaCl_2_, pH 7.4) containing MTT (1 mg/ml) for 90 min at 37°C in a humidified cell culture incubator. The resultant formazan crystals were solubilized in 450 µl of DMSO and two 195 µl aliquots were transferred to a 96-well plate. Absorbance was measured at 570 nm in a SpectraMax® i3x Multi-Mode Detection Platform (Molecular Devices, San Jose, CA). Percent cell viability was calculated by dividing the compound-mediated response by 100% cell viability, after subtraction of the background, and plotted as % cell viability versus log drug concentration.

## Results and discussion

This study contributes novel information on the interaction of TKI with equilibrative nucleoside and nucleobase transporters. Specifically, we a) characterized the uptake of 6-MP by ENBT1 in the K562 CML cell line, b) showed that TKI inhibit ENBT1 in low micromolar concentrations, and c) conducted a direct comparison of TKI inhibition of the two major subtypes of ENT (ENT1 and ENT2) expressed in the same cellular background. These results and their interpretation/significance are presented below. The full experimental data set can be viewed in S1 Table.

### Kinetics of 6-MP uptake by ENBT1 in K562 cells

**Fig 1:**
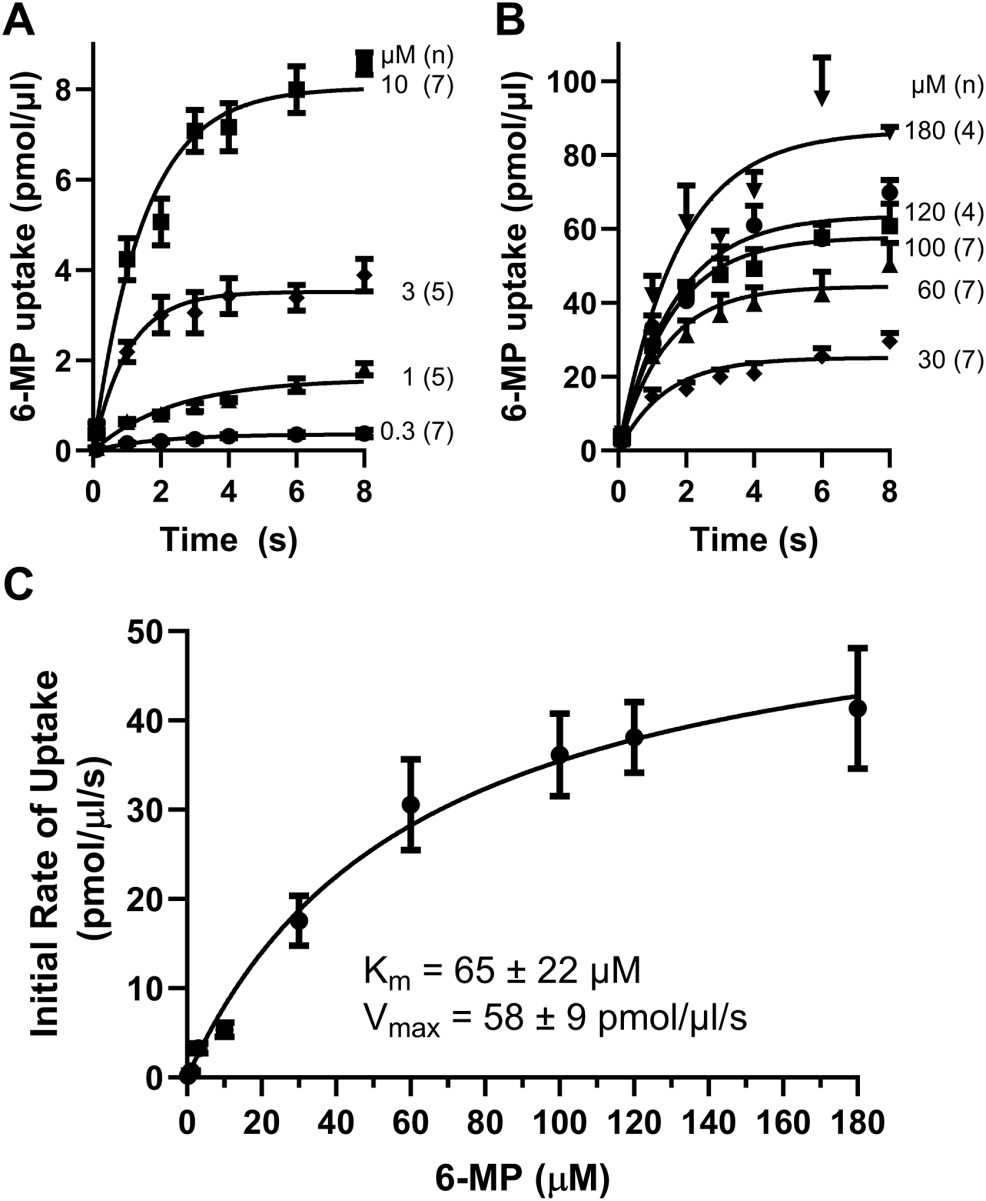
Uptake of [^14^C]6-MP by ENBT1 in K562 cells. Cells were incubated with a range of concentrations of [^14^C]6-MP (Panel A: 0.3–10 µM; Panel B: 30–180 µM) for 1-8 s, in the presence (non-mediated uptake) and absence (total uptake) of 5 mM adenine, and then processed to determine the amount of radiolabel accumulated by the cells. Data shown are the ENBT1-mediated uptake calculated as the difference between the total uptake and the non-mediated component. One-phase exponential association curves were fit to these data to determine the initial rate of uptake (estimated from the 100 ms time by extrapolation). The initial rates of influx determined for each [^14^C]6-MP concentration (pmol/µl cell water/s) were then plotted against the concentration of [^14^C]6-MP to determine the K_m_ and V_max_ for [^14^C]6-MP transport by ENBT1 in K562 cells **(C)**. Each point is the mean ± S.E.M. from the number of experiments (conducted in duplicate) indicated in parentheses on the figure.

The CML cell line K562 was used in this study as it represents a cancer type for which TKI are used therapeutically. K562 cells accumulated [^14^C]6-MP in an adenine-inhibitable process (ENBT1-mediated) so efficiently that it was necessary to construct time courses over a range of 6-MP concentrations to obtain initial rate of uptake estimates (as pmol/µl/100 ms; extrapolated from the curve-fit profiles shown in Fig 1A and B) that represent near zero-trans flux activity of the transport process. These initial rate estimates were used to construct a Michaelis Menten plot (Fig 1C) to obtain the K_m_ (65 ± 22 µM) and V_max_ (58 ± 9 pmol/µl/s) of 6-MP transport by the K562 cells. These values are similar to those reported previously for 6-MP transport by ENBT1 in several other leukemia cell lines [9].

### Inhibition of nucleoside/nucleobase transport by TKI

**Fig 2:**
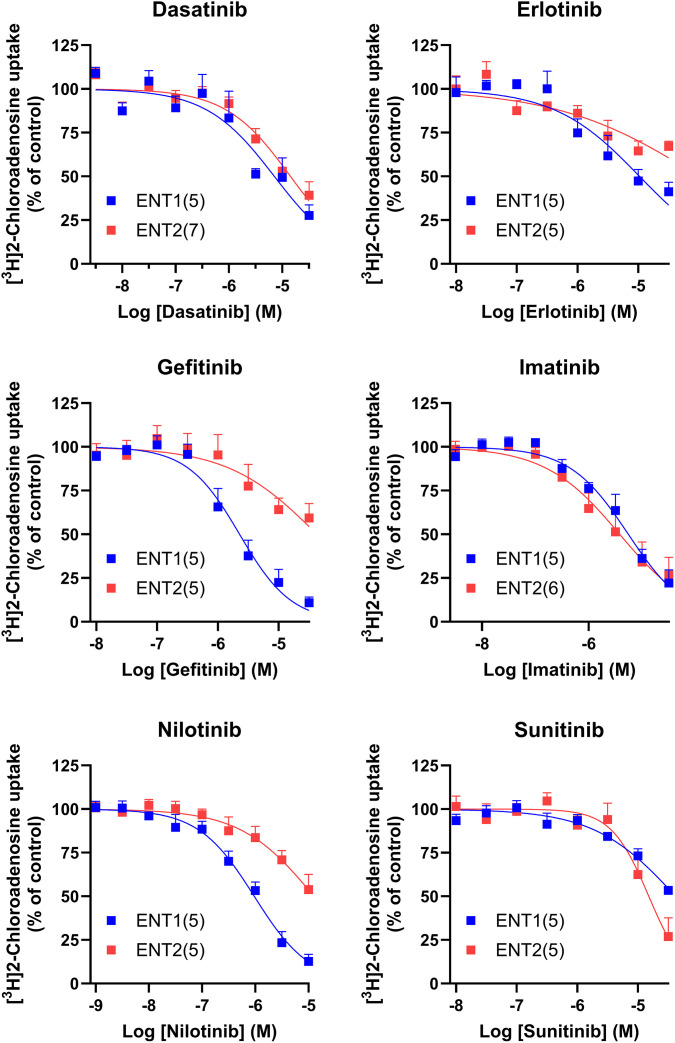
TKI inhibition of [^3^H]2-chloroadenosine uptake by ENT2KO cells (ENT1-mediated) and ENT1KO cells (ENT2-mediated). Cells were incubated for 15 s with 10 µM [^3^H]2-chloroadenosine and a range of concentrations of the indicated TKI. Cells were then separated from the media by centrifugation through an oil layer and processed to determine intracellular [^3^H] content. Data are presented as a percentage of the uptake observed in the absence of the inhibitor (Control). Each point is the mean ± S.E.M. from the number of independent experiments (n) shown in parentheses. Statistical analyses of the differences in K_i_ values for TKI inhibition of ENT1 versus ENT2, calculated from these data sets, are shown in Table 1.

**Fig 3:**
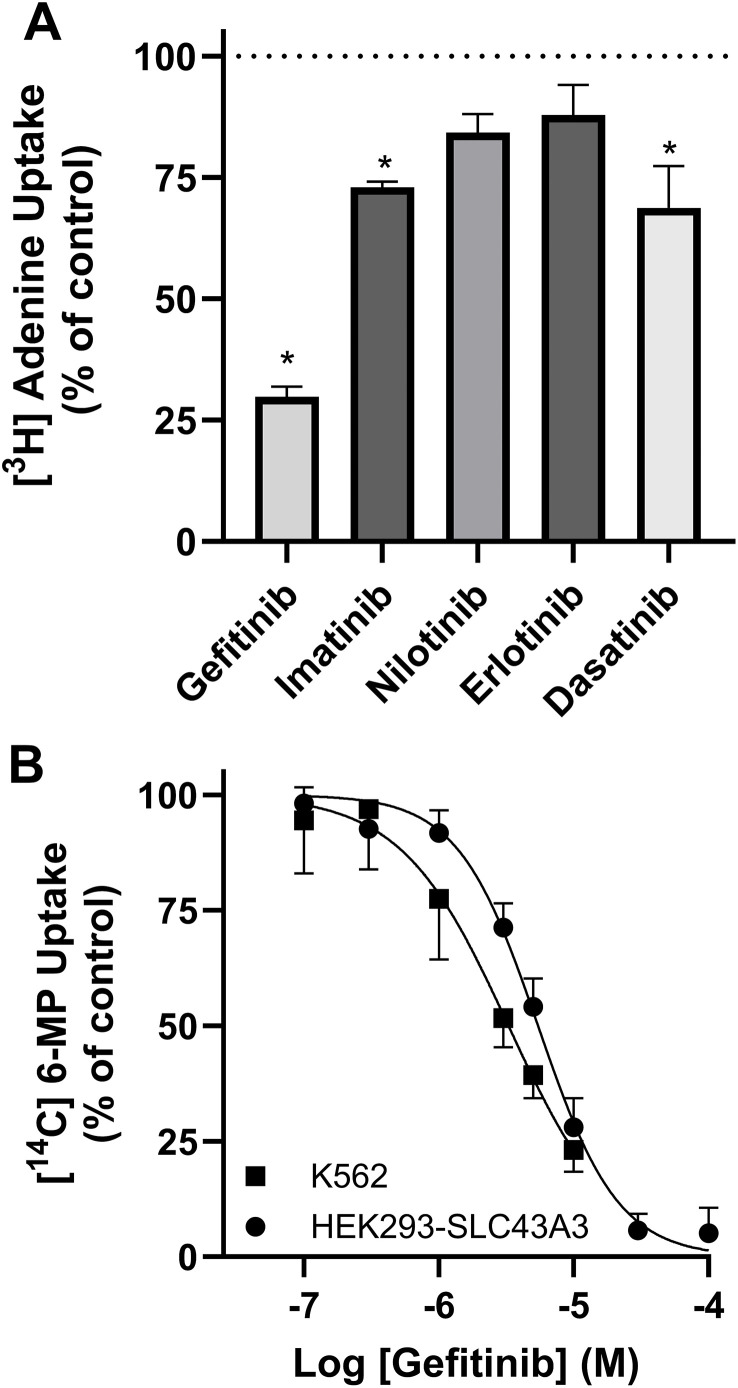
Inhibition of ENBT1 by TKI. **A)** The uptake of [^3^H]adenine by HEK293-SLC43A3 cells and un-transfected HEK293 cells was assessed in parallel using a 2 s time point in the presence and absence of 10 μM gefitinib, imatinib, nilotinib, erlotinib, and dasatinib. Data were normalized as a percentage of control uptake, where 100% was defined as the uptake of [^3^H]adenine by HEK293-SLC43A3 cells in the absence of inhibitor and 0% was defined as the uptake in un-transfected HEK293 cells. Each bar represents the mean ± S.E.M. of 6 experiments done in duplicate. * Indicates a significant difference from 100% control (one-way ANOVA with the post-hoc Holm-Šídák test, P < 0.05). **B)** A range of concentrations of gefitinib was assessed for the ability to inhibit the 2 sec uptake of 30 μM [^14^C]6-MP in HEK293-SLC43A3 and K562 cells. Data were normalized as a percentage of control uptake, with 100% defined as the uptake of 6-MP in the absence of inhibitor and 0% defined as the uptake in the presence of 5 mM adenine. Sigmoid curves were fitted to these data to determine IC_50_ values, which were then used to calculate the inhibitor K_i_ values presented in the text. Each point represents the mean ± S.E.M. of 5 (HEK293-SLC43A3) or 6 (K562) experiments done in duplicate.

The TKI tested inhibited ENT1-mediated transport of [^3^H]2-chloroadenosine with an order of affinity nilotinib > gefitinib > imatinib > dasatinib > erlotinib > sunitinib (Fig 2, Table 1), but all were within a narrow range of K_i_ values of 0.7 to 29 µM. This is similar to the affinities of TKI for ENT1 reported previously using lung and pancreatic cancer cell lines [12], as well as ENT1 expressed exogenously in yeast. [13] Nilotinib, gefitinib and erlotinib had significantly lower affinities for ENT2, compared with ENT1, while dasatinib, imatinib, and sunitinib had similar (not significantly different) affinities for both ENT subtypes (Table 1). With respect to the nucleobase transporter ENBT1, gefitinib, imatinib and dasatinib, screened at a single concentration of 10 µM, significantly inhibited the cellular uptake of the ENBT1 substrate [^3^H]adenine (Fig 3A, Table 1). However, only gefitinib produced sufficient inhibition to allow determination of a K_i_ value for inhibiting [^14^C]6-MP uptake by these cells, which, at 2.9 ± 1.4 µM, was similar to its affinity for inhibiting ENT1 (Fig 2B, Table 1). A similar affinity (K_i_ = 1.8 ± 0.9 µM) was obtained for gefitinib inhibition of [^14^C]6-MP uptake by ENBT1 in K562 cells, indicating that this effect is not cell type- or expression model-specific (Fig 3B). The ability of TKI to inhibit both nucleoside and nucleobase transporters with similar affinities suggests that they are interacting with a common element in each of these transporters, likely the purine ring interaction component of the substrate binding domain. This is consistent with the finding that TKI inhibit tyrosine kinase activity via interactions with the purine ring recognition domain of the ATP binding site of the enzyme. [3] It must be noted, however, that the design of this study does not allow one to determine the type of inhibition (competitive versus non-competitive) by TKI on these transporters. Nevertheless, this interaction suggests that these TKI have the potential to interfere with the uptake of nucleoside and nucleobase analogue drugs used as chemotherapeutic agents (e.g., 6-MP, gemcitabine) when used in combination therapies. However, the concentrations required to inhibit the transporters (>1 µM) are at the higher end of the range achieved with therapeutic doses of these drugs (see Table 1). Indeed, a search of the literature for reports of such interactions in the clinical use of such drug combinations did not reveal any noteworthy issues in this regard.

**Table 1:** Comparison of inhibition constants for TKI inhibition of ENT1, ENT2 and ENBT1.

	ENT1	ENT2	ENBT1	Plasma levels
	K_i_ (µM)^a^	K_i_ (µM) ^a^	% Inhibition at 10 µM ^b^	*Cmax (µM)* ^*d*^
**Nilotinib**	0.7 ± 0.3^c^	8.8 ± 6.1*	16 ± 4	2.9 [18]
**Gefitinib**	1.8 ± 0.7	>30*	69 ± 2	0.9 [19]
**Imatinib**	4.3 ± 1.6	2.7 ± 1.4	27 ± 1	3.2 [20]
**Dasatinib**	5.5 ± 3.0	9.9 ± 4.4	31 ± 8	0.3 [21]
**Erlotinib**	8.1 ± 6.0	35 ± 7*	12 ± 6	5.9 [22]
**Sunitinib**	29 ± 12	10 ± 4	–	0.13 [23]

a K_i_ values were derived from data shown in Fig 2

b Calculated from data shown in Fig 3A

c Each value is the mean ± S.E.M. from 5–7 experiments as detailed in the legends to Fig 2 and 3

d Calculated from plasma C_max_ data obtained in ng/ml at steady-state using typical therapeutic doses [referenced study].

* Significant difference between the K_i_ values for ENT1 and ENT2 (Students t-test, P < 0.05).

### Impact of ENT1, ENT2, and ENBT1 gain/loss on the effect of TKI on cell viability

**Fig 4:**
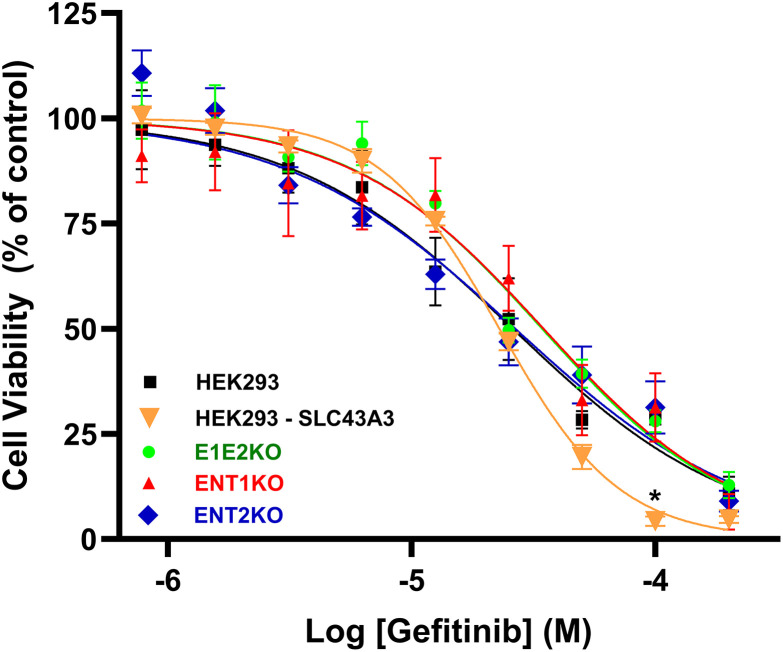
Effect of the absence or presence of ENT1, ENT2, or ENBT1 on the ability of gefitinib to decrease HEK293 cell viability. Unmodified HEK293 cells and the genetically modified ENT1KO, ENT2KO, E1E2KO cells, and *SLC43A3*-transfected HEK293 cells were plated in complete media and treated with a range of gefitinib concentrations for 48 h at 37°C. Cells were then assessed for viability using the MTT assay. Data are shown as mean ± S.E.M. from 7 (HEK293, ENT1KO, ENT2KO, E1E2KO) or 5 (HEK293-SLC43A3) independent experiments. * Indicates a significant difference from the HEK293 cells (Two-way ANOVA, P < 0.05, with the Dunnett post-test for multiple comparisons).

Compounds that interfere with substrate uptake via plasma membrane transporters can be either substrates themselves or non-transportable inhibitors that bind to the translocation domain of the proteins. TKI need to enter cells to interact with the ATP-binding domain of the tyrosine kinase receptors. If they are substrates for nucleoside or nucleobase transporters, removing one or more of these transporters from cells should reduce the intracellular accumulation of TKI and, consequently, their cytotoxicity. This was tested using gefitinib, a TKI with high affinity for all the transporters examined in this study. Gefitinib decreased HEK293 cell viability with a LogEC_50_ of −4.63 ± 0.06 (Fig 4). While the HEK293-SLC43A3 cells appeared to be slightly more sensitive to high concentrations of gefitinib, transfecting cells with *SLC43A3* (ENBT1), or removing *SLC29A1* (ENT1KO), *SLC29A2* (ENT2KO) or both (E1E2KO), had no significant impact on the overall ability of gefitinib to decrease cell viability (logEC_50_ values of −4.64 ± 0.02, −4.50 ± 0.08, −4.58 ± 0.05, and −4.47 ± 0.04, respectively). This suggests that none of these transporters are involved in TKI access to the intracellular milieu. This is compatible with a previous study which showed that, while the TKI loratinib was an effective inhibitor of ENT1 in HAP1 cells, the loss of ENT1 in those cells did not affect the intracellular concentrations of loratinib as measured by via mass-spectrometry [10]. The most effective TKI inhibitor of ENT1, gefitinib, as defined in the present study, was not, however, tested in that previous study; it may be of value to consider this analysis in future studies. Another approach would be to assess the ENT or ENBT1 mediated uptake of radiolabeled TKI analogues. This was attempted by our research group using [^3^H]gefitinib and the HEK293, HEK293-SLC43A3, and ENT2KO cell lines. However, a very high level of apparent non-specific binding of [^3^H]gefitinib to the cells precluded drawing any definitive conclusions regarding its cellular uptake from the experimental data obtained (unpublished observations).

### Lack of effect of gefitinib on 6-MP-mediated decrease in cell viability

**Fig 5:**
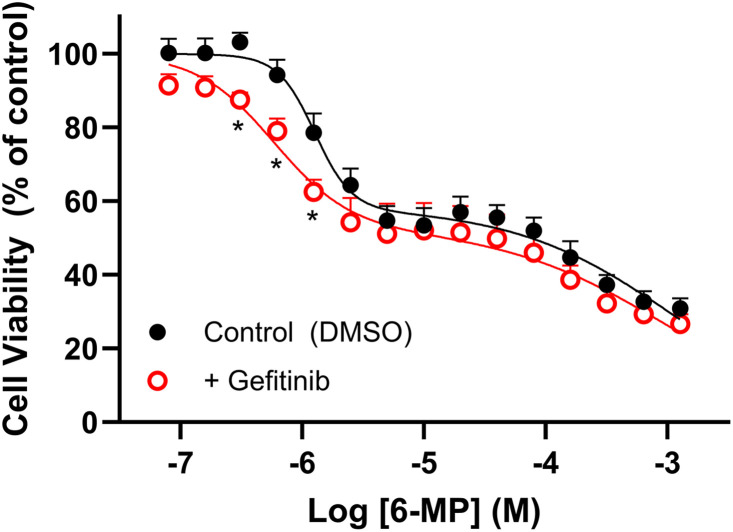
Effect of gefitinib on the ability of 6-MP to decrease cell viability. HEK293-SLC43A3 cells were plated in complete media and treated with a range of 6-MP concentrations for 48 h at 37°C in the absence (Control) or presence (+ Gefitinib) of 3 µM gefitinib. Cells were then assessed for viability using the MTT assay. Data are shown as mean ± S.E.M. from 6 independent experiments. The data set is best described as a biphasic inhibition profile with about 50% of the cell population showing sensitivity to 6-MP at concentrations of less than 1 µM. * Indicates a significant effect of the inclusion of gefitinib relative to control (two-way ANOVA, P < 0.05, with the Holm-Šídák post-test for multiple comparisons).

To further explore potential therapeutic interactions between gefitinib and the nucleobase transporter ENBT1, we examined the effect of gefitinib on the ability of 6-MP to decrease K562 cell viability. As noted in previous studies [9], 6-MP has a biphasic effect on cell viability, with approximately 50% of the cells being sensitive to 6-MP, characterized by an EC_50_ of 1.26 ± 0.23 µM (Fig 5). The inclusion of 3 µM gefitinib in this assay, a concentration that does not affect cell viability itself (Fig 4) but would produce about 30% inhibition of ENBT1 (Fig 3), significantly enhanced the effect of 6-MP on cell viability (EC_50_ of 0.65 ± 0.24 µM), suggestive of a synergistic drug interaction. This is opposite to what would be expected if gefitinib and 6-MP were competing for ENBT1 for cellular access. This synergy may reflect the reported ability of gefitinib to inhibit xanthine oxidase [24], thereby decreasing 6-MP metabolism to inactive metabolites.

## Conclusion

These data confirm and extend previous works suggesting that TKI interact with both of the major subtypes of equilibrative nucleoside transporters (ENT1 and ENT2) that are involved in the cellular uptake of chemotherapeutic nucleoside analogues. Furthermore, we have shown that the nucleobase transporter ENBT1 can also be inhibited by TKI at concentrations similar to those that inhibit nucleoside transporters. TKI likely compete with the binding of substrates to the purine ring recognition site of these transporters, but they do not appear to be substrates themselves as loss of these transporters does not affect the ability of TKI to reduce cell viability. While the potential exists for competition between TKI and purine analogues used in combination in chemotherapy regimens, the concentrations of TKI required to significantly inhibit these transporters make it unlikely that this is a significant source of drug-drug interactions for the compounds tested in this study when used at typical therapeutic doses.

## Supporting information

S1 Table Experimental data underlying Figures 1 through 5. (XLSX)
